# Supplementary material for: Hear it here: Built environments predict ratings and descriptions of ambiguous sounds
Source: PLoS One. 2025 Jan 15;20(1):e0316187. doi: 10.1371/journal.pone.0316187 (PMC11734970; doi:10.1371/journal.pone.0316187)
Supplement: Attachment — Submitted filename: author_rebuttal_rev1_final.docx [file pone.0316187.s001.docx]

We would like to thank the editor and reviewers for their thoughtful and constructive comments and their positive assessment of our manuscript. We have responded to all the suggestions, which we itemize below.

As a brief overview, our responses to the reviewers' comments involve several important additions. We strengthened the theoretical grounding of the Introduction, especially with regards to cognitive ethology and the priming literature. In the Methods section, we provided more details on the previous and current ratings of the auditory stimuli used. In the Results section, we conducted two mixed-effects models to control for the random effects of individual sounds, as well as a multivariate ANOVA to simultaneously predict bipolar ratings and sentiment scores.

Our responses to Editor and reviewer comments are given in blue**;** changes in the paper are in **bold** face.

Editor.

*Two experts in the field have carefully reviewed the interesting manuscript entitled “Hear it here: Built environments predict ratings and descriptions of ambiguous sounds“. You can find their comments below.*

*On my side, I would like also to have means and std of the ratings to see if the stimuli have remained neutral with wide dispersion or not.*

Thank you. We have now added a table (please see Table 2) with the means and SDs of our study’s bipolar scale ratings, alongside the original IADS valence and arousal ratings. These results show that, in our study, sounds were rated similarly close to the neutral point of the scale, and had a slightly smaller range of dispersion, compared to how they were rated in the original IADS norming of these sounds.

In light of these reviews and my own reading, I am requesting a major revision and resubmission, in which you will need to respond to each point in each review.

*Journal requirements:*

*When submitting your revision, we need you to address these additional requirements.*

*1. Please ensure that your manuscript meets PLOS ONE's style requirements, including those for file naming. The PLOS ONE style templates can be found at*

[*https://journals.plos.org/plosone/s/file?id=wjVg/PLOSOne_formatting_sample_main_body.pdf*](https://journals.plos.org/plosone/s/file?id=wjVg/PLOSOne_formatting_sample_main_body.pdf) *and*

[*https://journals.plos.org/plosone/s/file?id=ba62/PLOSOne_formatting_sample_title_authors_affiliations.pdf*](https://journals.plos.org/plosone/s/file?id=ba62/PLOSOne_formatting_sample_title_authors_affiliations.pdf)

We have now revised the manuscript’s formatting and naming scheme to match these style requirements.

*2. Thank you for stating the following financial disclosure:*

*“BJF: NSERC Canada Graduate Scholarship - Doctoral (CGS D) (569604)*

*AK: NSERC (170077-2011)*

*RMT: NSERC (F19-05182)”*

*Please state what role the funders took in the study.  If the funders had no role, please state: "The funders had no role in study design, data collection and analysis, decision to publish, or preparation of the manuscript."*

*If this statement is not correct you must amend it as needed.*

*Please include this amended Role of Funder statement in your cover letter; we will change the online submission form on your behalf.*

The funders had no role in study design, data collection and analysis, decision to publish, or preparation of the manuscript. We now state this in the revised cover letter along with a correction to AK's grant number: **NSERC (2022-03079)**.

Reviewers' comments:

Reviewer #1: It would be interesting, perhaps for a future publication, to have a more detailed description of the audio signals used as stimuli for the study.
Furthermore, some parts of the article repeat concepts already developed.

We appreciate this comment. We have now more thoroughly discussed the features of the auditory stimuli used for the study on page 6:

“The stimuli in our study were a series of **20** **audio recordings of everyday sounds, each** with a **uniform original duration of 6000** **ms (Table 2). We selected these** auditory stimuli from the International Affective Digitized Sounds system (IADS-2; [24]). **These sounds were presented at the same volume with no fading in or out.** All sounds selected had a mean rating between 5 and 5.9 (neutral point) on a **9**-point Self-Assessment Manikin scale rating from completely unhappy to completely happy [24], and all **but one sound ("Sink")** had a standard deviation **(SD)** in their pleasure rating of at least **1.5**.”

We have also indicated the specific sounds used from the IADS stimulus set, as well as the valence and arousal ratings provided in the original IADS study, in Table 2. In general, we have revised the manuscript to reduce repetition.

Reviewer #2: The submitted manuscript addresses an interesting question: does the immediate (built) environment (i.e., a modern vs an old room) influence valence judgements about neutral auditory stimuli (drawn from the IADS stimulus set). The authors consider additionally some qualitative data which is subject to sentiment analysis, and nested independent variables such as route taken to the room. Although, in a broad sense, the study is adequately put together and reaches a logical conclusion, there are a number of reasons why the study is not publishable at present. Below I present some quite detailed comments - I hope that they are helpful to the authors in formulating future iterations of the manuscript for resubmission to PLOS or elsewhere.

We thank the reviewer for their thoughtful comments and feedback, which we have sought to address below.

Keywords: embodied cognition is mentioned only once in the manuscript and isn't really used as the conceptual framework here. Valence scales and stimulus ratings aren't particularly informative. Also, this paper does not particularly take an ethological approach.

We have now revised our choice of keywords to better reflect the themes discussed in the paper, as follows: “Auditory psychology”, “Architectural psychology”, “Auditory perception”, and “Priming”.

Introduction:

The purpose of the article is clear. However, the literature review draws on all sorts of sources, and I think it could be more focused on environmental influences on auditory experience. There are some priming studies that consider cross-modal relationships between visual stimuli and sounds, e.g. Goerlich et al (2012), and there are plenty of others. Tadajua-Jiminez (2010), D'Alessandro et al (2018) might also be useful in the literature review. It will be really important to build a rationale for the study here - there is useful material in auditory perception, soundscapes and architecture literature - as it is, some of the points seem a bit tangential.

We have now revised the Introduction section (pages 2-5) to build a stronger rationale for environmental influences on auditory experience; cited and discussed the recommended priming studies as well as additional studies to support this rationale; and restructured the literature review such that it is more focused and cohesive.

If this phenomonen is conceptualised as transfer of affect from a valenced prime to a neutral target, the Affect Misattribution Procedure is probably the place to look for the cognitive underpinning of the effect - I was surprised not to see this mentioned, especially as priming is mentioned, albeit briefly, in the discussion.

We now discuss the Affect Misattribution Procedure, together with a more in-depth discussion of behavioural priming and transfer of valence from the environment to the auditory stimuli, in the Introduction on pages 2-3:

“**This transfer of valence between stimuli has previously been characterized through the Affect Misattribution Procedure (AMP) [7], an experimental paradigm capturing the misattribution of affective information to stimuli based on contextual information [8]. Thus, in a space with unwelcoming features, we may perceive a neutral or even pleasant sound as unpleasant [9] compared to if we heard that sound in a space with pleasant features. While past research has established cross-modal priming given the co-presentation of two stimuli with differing valences, we do not yet know whether we would see priming from more global environmental features to a specific stimulus.”**

We also discuss the AMP in the Discussion on pages 13-14:

“**As shown in the AMP [9], this information can prime attributions of the valence that participants assign to** stimuli they experience in the space - even if participants are unaware of the effects of the space on their sound ratings and descriptions. Thus, participant ratings of sounds could be proxies for their experience of the architectural context in which the sounds were heard - enhanced by the amount of time they spent moving through the space. **It is important to note that - contrary to earlier findings - recent evaluations of the AMP suggest that cross-modal priming is dependent on our awareness of being primed [36]. However, as participant perceptions of room pleasantness were not linked to sound ratings, it is reasonable to speculate that the effects observed in the present study were implicitly primed.**”

Also, there are some instances where the authors cite something but don't capture accurately what the cited article is saying, e.g., Son et al. (2015) isn't an auditory study and doesn't directly bear on the present manuscript.

If the reviewer is referring to our citation of Song et al. (2019), we have replaced references to this paper with more appropriate, auditory-focused references to Tajadura-Jiménez et al. (2010) and Wardhani et al. (2022) in the Introduction on page 3 and in the Discussion on page 15, respectively.

As with the keywords, the text leans on embodied cognition in the first paragraph, and claims to take an ethological approach later on - neither of these are really reflective of the manuscript as a whole.

We have revised the Introduction to remove the focus on embodied cognition in the first paragraph on page 2.

We have also introduced the ethological literature earlier and more thoroughly, on page 3:

**“The cognitive ethology approach [11–13] evaluates how our ability to complete various tasks is impacted by these tasks’ context. By focusing on how stimuli are situated and interpreted in their specific environments, we can better understand how the environment influences our perception of these stimuli.”**

There are some phrases that seem a little odd to me in academic writing, for instance "as such" is used four times. Additionally, there seems to be a mixture of registers in the writing. Most readers are happy with 'passive' scientific writing or with first person writing, but it would be better to stick with one or the other rather than mix.

We thank the reviewer for their feedback; we have revised the Introduction section to increase the variety of phrases used and revised the overall paper to use a consistent first-person register.

There is a lot of explanation of the benefits of rating scales, and the some variation phrase 'bipolar rating scales' is used several times. This reads more like a student trying to convince a marker that they know what they are doing, rather than something written for an interested and scientifically literate audience.

We have now revised the discussion of rating scales on pages 3-4 to be more concise and less repetitive to improve readability.

Methods:

Line 85 - there is more to sounds than frequencies. It really is the semantic-affective content of the IADS sounds that is important. Psychoacoustics (and frequencies are not the only part of this) is important as a determinant of affect, but not the only thing.
More details of the stimuli would be good - which IADS sounds did you use? what were the valence/arousal ratings in the original Bradley et al study. Were they uniformly 6000 ms or did you allow variation to allow for ecological validity of the sounds. What about volume? Were the onsets/offsets ramped/faded or did the sounds just start/stop?

We have now added additional information about the auditory stimuli used in the study. In Table 2, we list all sounds used and the original valence and arousal ratings reported for each sound from the IADS study. Furthermore, on page 6, we have now revised our description of the sounds to be more precise:

“The stimuli in our study were a series of **20** **audio recordings of everyday sounds, each** with a **uniform original duration of 6000** **ms (Table 2). We selected these** auditory stimuli from the International Affective Digitized Sounds system (IADS-2; [24]). **These sounds were presented at the same volume with no fading in or out.** All sounds selected had a mean rating between 5 and 5.9 (neutral point) on a **9**-point Self-Assessment Manikin scale rating from completely unhappy to completely happy [24], and all **but one sound ("Sink")** had a standard deviation **(SD)** in their pleasure rating of at least **1.5**.”

Additionally, we now mention in the Introduction on page 5 that the sounds did not have strong initial semantic-affective associations.

The main manipulation is the sites. Perhaps it would be worth including details of the sites in the method somewhere, e.g. a table comparing the two sites. Optionally, you could perhaps include photographs of the two sites?

In Table 3, we have now included details on the spatial configuration and characteristics of each site. In Figure 1, we have now included pictures of the study room at each site.

Line 104 - 0.4 seems like a sensible effect size to base power analysis on, but how did you arrive at it? Is it based on previous research?

We thank the reviewer for their feedback. On page 5, we have now clarified that this effect size was selected based on results from an online pilot study focusing on sound ratings for unipolar vs. bipolar scales, and are congruent with those observed in an existing study comparing sound ratings:

“We powered our study for a medium effect of 0.4 for a difference in bipolar sound ratings between sites, **based on the effect sizes observed in an online pilot study comparing ratings on unipolar vs. bipolar scales with the same stimulus set used in the present study. This effect size is also congruent with that found by Tajadura-Jiménez et al. [17] when looking at the effect of architectural features and sound position on perceived sound valence.**”

Overall, the procedure seems sound. Perhaps a schematic of the route*site*elevator design might be easier to digest than the text for some readers to understand the design quickly.

We thank the reviewer for their comments and have now included a schematic of the design in Figure 3.

Results:

I have some concerns about parts of the analysis. A simple t-test between old and new sites is not quite the right thing here. There needs to be some control for how participants are using the scale, and I can't see how the t-test can handle the different IADS sounds. One option is to normalise the ratings. An even better option would be something like a linear mixed effects model with rating~site+(1|Participant)+(1|Sound), which would be easy enough to implement in R (eg using lmer).

We have revised the Results section to replace the t-tests with the recommended linear mixed effects models (separated for predictors of sound ratings and sentiment scores), the results for which we now report in Tables 4 and 5, and described as follows on pages 9-10:

“**A linear mixed-effects model evaluating sound ratings by site, controlling for sound as a random effect,** revealed that participants rated all sounds as being significantly more pleasant in the New site compared to the Old site (Table 4, Figure 4A).”

“**A linear mixed-effects model evaluating sound sentiment scores by site, controlling for sound as a random effect,** revealed that the sentiment of sound descriptions did not significantly differ between sites (Table 5, Figure 4B).”

We have also updated the Methods section on page 8 to introduce this method:

**“**We compared bipolar scale and sentiment analysis ratings by site using **two mixed-effects linear models using the lmerTest package in R [32] predicting bipolar ratings and sentiment scores, respectively, from each site with participant and sound used as random effects.”**

Line 175 - a simple outline of how VADER calculates sentiment would be helpful to some readers, as there are a few different ways to calculate sentiment. From memory, the VADER GitHub has a neat description.

We thank the reviewer for their suggestion; we have now added a description of how VADER calculates sentiment on page 8:

**“VADER calculates sentiment scores by evaluating the valence of each word in each text response - accounting for the order of, and modifiers on, each word - to output a compound sentiment score for each participant’s response to each sound.”**

line 184: Bonferroni corrected for multiple comparisons - I can't really see where the multiple comparisons are. There are basically two separate t-tests.

In removing the t-tests from our analysis, we have also removed the Bonferroni correction for the p-values of the two t-tests.

Also, given that there are two correlated dependent variables, might manova remove some of the confusion?

To address this suggestion, we have now run an MANOVA to evaluate whether site, while controlling for sound, significantly predicts bipolar ratings and sentiment scores. We now introduce this method in the Methods section on page 8:

**“Additionally, to control for collinearity in bipolar scale and sentiment score results, we conducted a multivariate ANOVA using the manova function in R [33] with valence ratings and sentiment scores as distinct dependent variables.”**

We also report these results in the Results section on page 11:

**“Furthermore, a multivariate ANOVA analysis predicting bipolar scale ratings and sentiment scores revealed that, taken together, both measures significantly differed by site and by sound (Site: F(2, 1785) = 16.51, p <0.001, Pillai = 0.02; Sound : F(38, 3572) = 7.37, p <0.001, Pillai = 0.15).”**

Incidentally, the headings of the subsections refer to ratings but include analysis of the sentiments as well. I think the results section is short enough to be delineated by paragraphs rather the subsections.

We thank the reviewer for their recommendation; we have now removed the headings for the Results subsections.

Discussion:

'Nudge' is quite a specific concept in behavioural psychology. This should be introduced earlier or not at all, and discussed more fully if you use it. Same goes for 'priming' (line 252).

We have now integrated a discussion of both nudge and behavioural priming as underpinning behavioural decisions covered in the Introduction section on pages 2-3. In particular, we have indicated how information from environmental features could nudge participants to make decisions, as well as prime the attribution of specific valence types to stimuli experienced in those environments:

“**To this end, past** studies have identified ways in which spaces with distinct features can **nudge us towards making specific behavioural decisions through subtle incentives and cues [14,15].”**

**“**The general representation of the built environment could impact how we appraise stimuli within it **through a process of behavioural priming, such that an environment with more positively valenced features could prime positive stimulus ratings - with negative features priming negative ratings [4].”**

Line 233/236 - the switch to 'aversive' from negative seems like you're talking about something slightly different from before. It would be easier for the reader if you were consistent in terminology.

We have revised all mentions of “aversive” in the paper to refer to “negative valence” to ensure consistent terminology. The one exception is on page 14 where aversiveness qualifies a discussion of negative valence.

As with the Introduction, the Discussion hops around a lot of areas. I'd suggest keeping it really focused on environmental/architectural influences on affective ratings of auditory stimuli. This has implications for instance in theatre design, and is interesting as a question in basic science.

We thank the reviewer for their feedback and have streamlined the Discussion section to focus it more on environmental and architectural impacts on auditory stimuli ratings. We have also integrated a discussion of the implications of the findings for theatre design and basic science on page 14:

**“These results could also have implications for a wide variety of fields, including designing theatre sound effects and music to elicit specific emotional responses in audiences and maximize the impact of a performance [40,41].”**

Finally, I hope the authors are not disheartened by the review - this is an interesting manuscript, and has the potential to contribute to architectural/auditory psychology.

Thank you for your helpful and constructive comments. We believe that they have greatly improved the manuscript.
