# Supplementary material for: Hear it here: Built environments predict ratings and descriptions of ambiguous sounds
Source: PLoS One. 2025 Jan 15;20(1):e0316187. doi: 10.1371/journal.pone.0316187 (PMC11734970; doi:10.1371/journal.pone.0316187)
Supplement: Attachment — Submitted filename: author_rebuttal_rev2.docx [file pone.0316187.s002.docx]

We would like to once again thank the editor and reviewers for their thoughtful and constructive comments and recommendations to our manuscript. We have addressed this additional comment below and feel that the manuscript is stronger as a result of their feedback.

In our response to the editor’s comments, we now discuss the differences in the results given by the linear mixed models and the MANOVA, with regards to potential differences in how well each model accounts for collinearity. Responses to the editor’s comments are given in blue**;** changes in the paper are in **bold** face.

We address the editor’s comment below.

*Dear authors, thanks for your careful attention to reviewers' comments; I find the manuscript has greatly improved.*

*There is still one point that is confusing for me: linear mixed models and MANOVA give different results with respect to the significance of sentiment description differences between both sites. However, in the abstract and discussion you state that there were no differences. Please clarify this choice.*

We thank the editor for their suggestions. We have now revised the abstract and discussion section to explain the differences in the MANOVA and linear mixed model results. The linear mixed effect models did not account for potential collinearities in the regressors used, while the multivariate ANOVA was able to capture the contribution of site and sound identity to both bipolar ratings and sentiment levels. The changes in the abstract are as follows:

“Participants rated sounds as being more pleasant at the New site compared to the Old site, but the sentiment of their descriptions **only differed between sites when controlling for collinearity**”.

The changes in the discussion section are as follows. On page 10:
“When participants described how they felt about the sounds, the valence of the sentiment of these descriptions **only differed between sites in our multivariate ANOVA analysis - which controlled for collinearity - but not in our linear mixed model analyses. Participants thus used moderately more negatively valenced language to describe sounds at the older site compared to the newer site.**”

On pages 11-12:

“The attribution of differences in sound interpretation to the site in which they were heard may be challenged by the lack of difference in sentiment ratings between sites in participant descriptions of sounds **in our linear mixed model analyses**.”

“In spite of our finding that only bipolar sound ratings differed between sites **with linear mixed model analyses**, sentiment scores were significantly correlated with bipolar ratings - a result supported by our multivariate ANOVA analysis showing that site predicted both sound ratings and sentiment scores. **This MANOVA result’s contrast with the non-significant linear mixed model results could speak to how the linear mixed models do not account for collinearity among bipolar ratings and sentiment scores, thus weakening the power of this analysis to detect differences between aspects of the auditory experience by site as compared to the MANOVA.**”
